# Regular physical activity and mammographic density: a cohort study

**DOI:** 10.1007/s10552-018-1075-3

**Published:** 2018-09-07

**Authors:** Shadi Azam, Katja Kemp Jacobsen, Arja R. Aro, My von Euler-Chelpin, Anne Tjønneland, Ilse Vejborg, Elsebeth Lynge, Zorana J. Andersen

**Affiliations:** 1 Unit for Health Promotion, Department of Public Health, University of Southern Denmark, Niels Bohrs Vej 9, Esbjerg, Denmark; 2 Department of Technology, Faculty of Health and Technology, University College Copenhagen, Copenhagen, Denmark; 3 Section of Environmental Health, Department of Public Health, University of Copenhagen, Copenhagen, Denmark; 4 Danish Cancer Society Research Center, Danish Cancer Society, Copenhagen, Denmark; 5 Department of Radiology and Diagnostic Imaging Centre, Copenhagen University Hospital, Rigshospitalet, Copenhagen, Denmark

**Keywords:** Mammographic density, Physical activity, Breast density

## Abstract

**Purpose:**

Physical activity is a modifiable lifestyle risk factor in prevention of breast cancer. Mammographic density (MD) is a strong risk factor for breast cancer. We investigate the association of regular physical activity with MD.

**Methods:**

For 5,703 women who participated in the Danish Diet, Cancer and Health cohort (1993–1997) and attended mammographic screening in Copenhagen (1993–2001), MD was assessed at the first screening after cohort entry. MD was defined as a binary measure equivalent to Breast Imaging Report and Data System (BI-RADS) to either mixed/dense or fatty. Participation and duration in physical activities (hours/week) and confounders were assessed by questionnaire at cohort baseline. Logistic regression was used to estimate associations [odds ratios (OR), 95% confidence intervals (CI)] between physical activities and MD.

**Results:**

56.3% of women had mixed/dense MD and 47.6% participated in sports. We found a significant positive association between participation in sports (OR 1.15; 95% CI 1.03–1.28) and do-it-yourself work (1.17; 1.05–1.31) and odds of having mixed/dense MD, which attenuated (1.08; 0.96–1.22 and 1.11; 0.98–1.25, respectively) in a fully adjusted model. No associations were found for time spent on physical activities or total metabolic equivalent of task scores with MD, in fully adjusted models. There was no effect modification of association between any physical activities and MD by obesity (BMI ≥ 30 kg/m^2^) and menopause status.

**Conclusions:**

Physical activity is not a determinant of MD.

**Electronic supplementary material:**

The online version of this article (10.1007/s10552-018-1075-3) contains supplementary material, which is available to authorized users.

## Introduction

Physical activity is recognized as a modifiable lifestyle risk factor in primary prevention of breast cancer. It is well established that regular physical activity is associated with a reduced risk of breast cancer [1–6]. A meta-analysis found that physically active women have 12% reduced risk of breast cancer compared to the inactive women [7].

Physical activity been hypothesized to reduce breast cancer risk through several mechanisms, including weight loss, obesity prevention, reduced sex hormone exposure, reduced levels of insulin and insulin-like growth factor exposure, induced immune system function, and mechanism of DNA repair [8]. Some of these factors, such as alteration in the metabolism of endogenous hormones, are suggested to influence mammographic density (MD) [8].

MD is increasingly being used as a biomarker of breast cancer risk, as it is one of the strongest risk factors [9]. MD refers to the amount of radiologically dense breast consisting of epithelial or stromal tissue that appears light on a mammogram, whereas fat tissue appears dark on a mammogram [10]. The density of the breasts can be measured qualitatively according to the Breast Imaging Reporting and Data System (BI-RADS) or quantitatively as the percent mammographic density (PMD). Women of same age and body mass index (BMI), with very dense breasts (> 75% density in the breast), have a four to six times greater risk of breast cancer than women with little density (10–5% density) or fatty breasts [11].

A number of studies on the association between physical activity and MD have reported inconsistent results [12–25]. Most studies found no association between physical activity and MD [20, 21, 23–25], whereas some reported an inverse association [12, 14, 22]. Three studies found that the inverse association was more pronounced in obese (body mass index (BMI) ≥ 30 kg/m^2^) and overweight (BMI 25.0–29.9 kg/m^2^) postmenopausal women [13, 15, 17]. Furthermore, few studies detected a weakly positive association between physical activity and MD, which was attenuated after adjustment for BMI [16, 18, 19]. The purpose of this study is to evaluate the association of leisure, transport-related, and occupational physical activity with MD for the first time in Danish population of women participants of the Danish Diet, Cancer and Health (DCH) cohort.

## Population and methods

### Danish Diet, Cancer and Health cohort

Between 1993 and 1997, a total of 160,725 persons (72,729 women), 50–64 years of age, born in Denmark, living in Copenhagen or Aarhus (the two largest cities in Denmark), and with no record of cancer in the Danish Cancer Registry, were invited to participate in the Danish prospective diet, cancer and health (DCH) cohort. In total 57,053 people, of whom 29,875 were women (37% of invited women and 7% of the entire Danish female population in this age group), accepted the invitation and participated in the study and answered a detailed questionnaire on diet, health, education, occupation, history of diseases, and other health-related items. Waist circumference (cm), height (cm), and weight (kg) were measured at the day of recruitment and BMI (weight^2^/height) was calculated. A detailed description of the DCH cohort has been published previously [26].

### Study cohort

The study sample consists of 5,703 (1,202 pre- and 4,501 postmenopausal) women above the age 50 years who participated in DCH cohort between 1993 and 1997 and attended the Copenhagen mammography screening program between 1993 and 2001.

### Physical activity assessment

Physical activity information was collected by a self-administered, interviewer-checked questionnaire in which leisure time and utilitarian transport-related (traveling to and from work, shopping, etc., for walking and cycling) physical activity was reported as hours/week spent on sports, cycling, gardening, walking, housework, and ‘do-it-yourself’ activities. Information was collected separately for winter and summer of the previous year, and the two values were averaged, so that being active implies at least half of an hour spent on a specific activity per week. The physical activity questions have been validated [27] and categorized into four levels (no activity, 0.5–2.0 h/week, 2.0–4.0 h/week, and ≥ 4 h/week). The total energy per week spent on leisure time physical activity was evaluated using the metabolic equivalent (MET) score, defined as the ratio of working metabolic rate to a standard metabolic rate, where one MET corresponds to resting metabolic rate obtained during quiet sitting [28]. To calculate the summed MET score, the time spent on each leisure time activity was weighted with a scalar according to the presumed intensity of the activity and summed for all activities [29]. According to the Compendium of physical activities [30], the following MET values were used: walking 3.0, cycling 6.0, gardening 4.0, sports 6.0, do-it-yourself work 4.5, and household work 3.0. Physical activity at work was assessed in five categories: sedentary (mainly sitting), standing, light manual, heavy manual, or unemployed. Separate questions on physical activity at work were assessed in five categories: sedentary, standing, light manual, heavy manual, or unemployed.

### MD measurement

The Copenhagen mammography screening program started in 1991 and targeted about 40,000 women aged 50–69 years at the start of each biennial invitation round resulting in 134,640 women who participated in screening between 1991 and 2001 [31, 32]. In this study, we included women who participated in Copenhagen mammography screening and in DCH cohort, and chose the single MD reading closest to, but subsequent to the cohort baseline in 1993–1997, when physical activity data were assessed. One radiologist was in charge of the screening which took place at a single Copenhagen hospital. All screens were taken by radiographers or X-ray nurses and evaluated independently by two trained radiologists who did not meet the attending women, unless they were recalled for assessment. Two-view mammography was taken on the first screen, on both breasts, a craniocaudal and oblique. MD is defined as a binary measure of either as fatty breasts, equivalent to Breast Imaging Reporting and Data System (BI-RADS, Atlas, 2008) density code 1 and part of code 2, or as mixed/dense breasts, equivalent to part of BI-RADS code 2, 3, or 4. Women with a negative screening and fatty breasts were scheduled to have only an oblique view of both breasts at their next screening, whereas women with a negative screening test and mixed/dense breasts were scheduled for two-view mammography of both breasts at the next screening. To evaluate MD readings, two radiologist had to come to an agreement on the MD readings. In cases in which readings did not read consensus, the evaluation was send to a third radiologist. We could not estimate inter-reader agreement in this study sample, but very experienced readers have generally high inter-observer agreement in Copenhagen mammography screening program, as documented earlier. This internationally unique dichotomous outcome for MD has been successfully validated showing expected associations with the BI-RADS density classification system with a substantial agreement with inter-rater variability (weighted kappa statistics) of 0.75 [33]. In addition, the Danish dichotomous MD score has been utilized in several earlier studies, showing the expected doubling of breast cancer risk in women with mixed/dense as compared to those with fatty breasts [34], as well as showing strong inverse association with BMI in children [35], inverse association with active smoking in adult age [36], no association with air pollution [37], significant association with higher MD among women with high alcohol consumption in early adulthood [38], and positive association between current use of HRT with MD and breast cancer risk [39]. We linked the Copenhagen mammography register to the DCH cohort by using the personal identification (CPR) number of the Danish Civil Registration System [40]. We used MD assessed at the first screening after the cohort baseline (1993–1997).

### Statistical methods

Logistic regression was used to estimate the association of MD with participation (yes, no) in six domains of physical activity (participation in sports, walking, cycling, gardening, do-it-yourself work, and housework), time spent on physical activities (no activity, 0.5–2 h/week, 2–4 h/week, and ≥ 4 h/week), and occupational physical activity (sedentary, standing, light manual, heavy manual, unemployment). Effects of physical activity were estimated in four steps: (1) crude model, adjusted for age, (2) a model additionally adjusted for the following a priori selected confounders: alcohol intake (g/day), menopause, hormone replacement therapy (HRT) use, HRT duration, number of children, previous benign tumor, age at first birth, smoking (never, previous, current), and education (short education ≤ 7 years, medium education: 8–10 years, long education: > 10 years), and mutual adjustment for other physical activities and occupational physical activity (e.g., to investigate the association between sport and MD we adjusted for cycling, walking, gardening, housework, do-it-yourself work, and for occupational physical activity), and (3) a model additionally adjusted for BMI and waist circumference. To test the effect modification of an association between physical activities and MD by obesity and menopausal status, analyses were conducted separately and in combination for women with BMI ≥ 30 and < 30 kg/m^2^ and for premenopausal and postmenopausal women. Analyses were performed in Stata 14 with logistic function, and presented as odds ratios (OR’s) and 95% confidence intervals (CIs). As a sensitivity analyses, we ran relative regression analyses, with glm function (binomial family and log link) in Stata, and presented results as relative risks (RRs) and 95% CIs.

The study was based on the registered and cohort data and was approved by Danish Data Protection Agency. We have obtained informed consent from all the participating women that information about them in health registries can be obtained.

## Results

Compared to the entire cohort, participants of this study were older, more likely to be nulliparous, HRT users, overweight, physically inactive, and less educated (online source 1). The mean time between recruitment and mammographic screening at which MD was assessed was 1 year. The mean age at the cohort baseline was 56.2 years, and the majority of women (56.3%) had mixed/dense breasts (Table 1). Women with mixed/dense breasts were younger, had lower BMI, lower waist circumference, were more likely premenopausal and HRT users, were more likely to have had previous benign tumor, to be nulliparous, had lower mean number of children, higher alcohol consumption, and had higher education level than women with fatty breasts.

**Table 1:** Characteristics of 5703 participants in the Danish Diet, Cancer and Health cohort by mammographic density (MD) assessed in the Copenhagen Mammography Screening Program

	Total	Mixed/dense	Fatty	*p* values^a^
		MD	MD	
Participants, *n* (%)	5,703	3,212 (56.3)	2,491 (43.7)	
Age (years), mean (SD)	56.2 (4.6)	55.4 (4.2)	57.34 (4.4)	0.0001*
BMI (kg/m^2^), mean (SD)	25.9 (4.6)	24.61 (3.9)	27.57 (5.0)	0.0001*
BMI < 18.5 (kg/m^2^),* n* (%)	77 (1.4)	62 (1.9)	15 (0.6)	0.000*
BMI 18.5–24.9 (kg/m^2^), *n* (%)	2,713 (476)	1,889 (58.8)	824 (33.1)	
BMI 25.0–29.9 (kg/m^2^),* n* (%)	1,946 (34.1)	954 (29.7)	992 (39.8)	
BMI ≥ 30 (kg/m^2^),* n* (%)	967 (17.0)	307 (9.5)	660 (26.5)	
Waist circumference (cm)	82.4 (11.8)	79.0 (10.1)	86.7(12.3)	0.0001*
Premenopausal	1,202 (21.1)	768 (23.9)	434 (17.4)	0.000*
Postmenopausal	4,501 (78.9)	2,444 (76.0)	2,057 (82.5)	
HT ever used,* n* (%)	2,725 (47.9)	1.644 (51.4)	1,081 (43.6)	0.000*
HT never used,* n* (%)	2,955 (52.0)	1,555 (48.6)	1,400 (56.4)	
Hormone therapy duration among ever users (years), mean (SD)	2.81 (5.0)	3.0 (5.1)	2.57 (4.9)	0.0001*
Previous benign tumor	745 (13.3)	530 (16.5)	215 (8.6)	0.000*
Nulliparous,* n* (%)	842 (14.8)	563 (17.5)	279 (11.2)	0.000*
Mean (SD) number of children*	1.84 (1.15)	1.69 (1.1)	2.0 (1.2)	0.0001
Mean (SD) age at first birth (years)	18.58 (9.4)	18.18 (9.9)	19.0 (9.7)	0.5750
Does not consume alcohol,* n* (%)	197 (3.5)	101 (3.1)	96 (3.9)	0.165
Does consume alcohol,* n* (%)	5,506 (96.5)	3,111 (96.9)	2,395 (96.1)	
Alcohol consumption in alcohol consumers (g/day), mean (SD)	5692 (13.3)	14.3 (16.5)	12.1 (16.3)	0.0001*
Never smoker	2,073 (36.5)	1,173 (36.6)	900 (36.4)	0.600
Previous smoker	1,253 (22.1)	691 (21.6)	562 (22.7)	
Current smoker	2,350 (41.4)	1,336 (41.8)	1,014 (41.0)	
Short education (≤ 7 years),* n* (%)	2,058 (36.0)	1.005 (31.3)	1,053 (42.3)	0.000*
Medium education (8–10 years),* n* (%)	2778 (48.7)	1614 (50.3)	1164 (46.7)	
Long education (> 10 years),* n* (%)	867 (15.2)	593 (18.5)	274 (11.0)	
Physically active,* n* (%)				
Participation in sports	2,710 (47.6)	1,582 (49.3)	1,128 (45.3)	0.003*
Walking	5,283 (92.7)	2,984 (93.0)	2,299 (92.4)	0.411
Cycling	4,003 (70.2)	2,306 (71.8)	1,697 (68.1)	0.003*
Gardening	2,979 (52.2)	1,700 (52.9)	1,279 (51.3)	0.240
Housework	5,615 (98.6)	3,166 (98.7)	2,449 (98.4)	0.474
Do-it-yourself work	2,225 (39.1)	1,333 (41.5)	892 (35.9)	0.000*
Physical activity h/week, mean (SD)				
Participation in sports	1.07 (1.8)	1.1 (1.9)	1.0 (1.7)	0.0125
Walking	5.51 (6.5)	5.3 (6.3)	5.6 (6.7)	0.1684
Cycling	3.0 (4.0)	3.1 (4.0)	3.0 (4.1)	0.1280
Gardening	1.67 (2.9)	1.65(2.9)	1.70 (2.8)	0.9830
Housework	7.4 (6.5)	7.1 (6.3)	7.7 (6.9)	0.0031*
Do-it-yourself work	0.98 (2.4)	1.03 (2.5)	0.91 (2.3)	0.0007*
Occupational activity,* n* (%)				
Sitting	2,174 (38.1)	1,288 (40.1)	886 (35.6)	0.0001*
Standing	994 (17.4)	561 (17.48)	433 (17.4)	
Light manual	1,255 (22.0)	739 (23.03)	516 (20.7)	
Heavy manual	119 (2.0)	64 (2.0)	55 (2.2)	
Unemployment	1,155 (20.2)	557 (17.3)	598 (24.0)	
MET score, mean (SD)	77.5 (53.2)	77.0 (51.6)	78.1 (55.1)	0.059

*SD* standard deviation, *BMI* body mass index, *MET* metabolic equivalent of task, *HT* hormone therapy

*p values < 0.05

^a^ *p* values were calculated with Kruskal–Wallis test for continuous values and Pearson’s *X*^2^-test for categorical values

Women with mixed/dense MD were more likely to participate in sports, cycling, and do-it-yourself work, but the energy spent on total activity as measured by the MET score was almost identical in women with fatty and mixed/dense breasts. Compared to women with fatty breasts, more women with mixed/dense MD had a sedentary occupation, and fewer were unemployed.

47.6% participated in sports, 92.7% walking, 70.2% cycling, 52.2% gardening, 98.6% housework, and 39.1% do-it-yourself work (Table 1). In the crude models we found a significant positive association between participation in sports (OR 1.15; 95% CI 1.03–1.28), cycling (1.09; 95% CI 0.97–1.31), do-it-yourself work (OR 1.05; 95% CI 1.05–1.31), and odds of having mixed/dense MD (Table 2). In the fully adjusted models, there was a weak, statistically insignificant association between participation in sports (OR 1.08; 95% CI 0.96–1.22), cycling (OR 1.08; 95% CI 0.95–1.23), gardening (OR 1.04; 95% CI 0.93–1.17), do-it-yourself work (OR 1.11; 95% CI 0.98–1.25) and MD, and none with walking (OR 0.99; 95% CI 0.79–1.24). There was a weak inverse association between housework (OR 0.89; 95% CI 0.53–1.49) and MD (Table 2). Further adjustment for BMI and weight circumference lowered the OR slightly, for all physical activities. There was no indication of a dose–response relationship between time spent on any of the activities and MD. However, after adjustment for BMI and waist circumference, we found a statistically non-significant inverse association with MD in women participating ≥ 4 h/week in sports, walking, cycling, and housework. There was association between total MET score or occupational physical activity and MD (Table 2). There was no effect modification of association between any of the physical activities and MD by obesity (BMI ≥ 30 kg/m^2^) or menopausal status separately (Tables 3, 4) and in combination (Table 5). We could not run relative regression analyses for fully adjusted models due to convergence problems. However, we ran crude and age-adjusted models, and as expected, found that associations estimated with relative regression analyses were attenuated (online source 2) as compared to those estimated by logistic regression (Table 2), strengthening the conclusion that there is a null association between physical activity and MD.

**Table 2:** Association of physical activity with MD among 5703 women in Diet, Cancer and Health cohort

	Mixed/dense	Fatty	Crude	Age adjusted	Fully adjusted^a^	Fully adjusted^a^ (+ BMI and waist circumference)
	MD	MD	OR (95% CI)	OR (95% CI)	OR (95% CI)	OR (95% CI)
Physical activity						
No participation in sport	1,627	1,360	1	1	1	1
Participation in sport	1,582	1,128	1.17 (1.05–1.30)	1.15 (1.03–1.28)	1.08 (0.96–1.22)	1.01 (0.89–1.14)
No walking	225	189	1	1	1	1
Walking	2,984	2,299	1.09 (0.89–1.33)	1.03 (0.85–1.27)	0.99 (0.79–1.24)	0.91 (0.72–1.16)
No cycling	906	794	1	1	1	1
Cycling	2,306	1,697	1.19 (1.06–1.33)	1.09 (0.97–1.23)	1.08 (0.95–1.23)	0.97 (0.85–1.11)
No gardening	1,512	1,212	1	1	1	1
Gardening	1,700	1,279	1.06 (0.95–1.18)	1.07(0.96–1.19)	1.04 (0.93–1.17)	1.03 (0.91–1.16)
No do-it-yourself work	1,876	1,596	1	1	1	1
Do-it-yourself work	1,333	892	1.27 (1.14–1.42)	1.17 (1.05–1.31)	1.11 (0.98–1.25)	1.06 (0.93–1.21)
No housework	43	39	1	1	1	1
Housework	3,166	2,449	1.17 (0.78–1.81)	1.08 (0.69–1.69)	0.89 (0.53–1.49)	0.89 (0.51–1.55)
Physical activity h/week						
Participation in sport						
No activity	1,627	1,360	1	1	1	1
0.5–2.0	800	572	1.17 (1.03–1.33)	1.15 (1.00–1.31)	1.10 (0.95–1.26)	1.02 (0.88–1.18)
2.0–4.0	546	379	1.20 (1.04–1.40)	1.17 (1.00–1.37)	1.11 (0.95–1.31)	1.02 (0.86–1.21)
≥ 4	236	177	1.11 (0.90–1.37)	1.12 (0.90–1.39)	1.06 (0.85–1.33)	0.95 (0.75–1.20)
*p* trend^b^						0.015
Walking						
No activity	225	189	1	1	1	1
0.5–2.0	522	374	1.17 (0.92–1.48)	1.09 (0.86–1.39)	1.04 (0.80–1.35)	0.98 (0.75–1.29)
2.0–4.0	897	669	1.12 (0.91–1.40)	1.04 (0.84–1.31)	0.96 (0.75–1.22)	0.90 (0.70–1.16)
≥ 4	1,565	1,256	1.05 (0.85–1.29)	1.02 (0.82–1.26)	1.00 (0.79–1.25)	0.90 (0.70–1.15)
*p* trend^b^						0.111
Cycling						
No activity	906	794	1	1	1	1
0.5–2.0	634	433	1.28 (1.09–1.50)	1.22 (1.04–1.43)	1.16 (0.98–1.38)	1.07 (0.90–1.28)
2.0–4.0	673	494	1.19 (1.03–1.39)	1.11 (0.95–1.29)	1.09 (0.93–1.29)	0.97 (0.82–1.16)
≥ 4	999	770	1.14 (0.99–1.30)	1.01 (0.88–1.16)	1.02 (0.88–1.19)	0.91 (0.77–1.06)
*p* trend^b^						0.537
Gardening						
No activity	1,512	1,212	1	1	1	1
0.5–2.0	724	504	1.15 (1.00–1.32)	1.05 (0.96–1.26)	1.05 (0.90–1.22)	1.01 (0.86–1.18)
2.0–4.0	481	372	1.04 (0.89–1.21)	1.05 (0.90–1.23)	1.05 (0.88–1.24)	1.07 (0.89–1.27)
≥ 4	495	403	0.98 (0.84–1.15)	1.05 (0.90–1.23)	1.03 (0.87–1.22)	1.02 (0.86–1.22)
*p* trend^b^						0.642
Do-it-yourself work						
No activity	1,876	1,596	1	1	1	1
0.5–2.0	789	513	1.31 (1.15–1.49)	1.20 (1.06–1.38)	1.13 (0.98–1.30)	1.07 (0.93–1.25)
2.0–4.0	325	225	1.23 (1.02–1.48)	1.11 (0.92–1.33)	1.06 (0.87–1.30)	1.02 (0.83–1.26)
≥ 4	219	154	1.21 (0.97–1.50)	1.13 (0.91–1.41)	1.09 (0.86–1.38)	1.06 (0.83–1.36)
*p* trend^b^						0.069
Housework						
No activity	43	39	1	1	1	1
0.5–2.0	141	96	1.23 (0.80–2.21)	1.24 (0.74–2.07)	0.99 (0.56–1.77)	0.97 (0.52–1.82)
2.0–4.0	814	582	1.27 (0.81–1.98)	1.15 (0.73–1.82)	0.92 (0.55–1.55)	0.88 (0.50–1.56)
≥ 4	2,211	1,771	1.13 (0.73–1.75)	1.05 (0.67–1.64)	0.89 (0.53–1.48)	0.87 (0.50–1.53)
*p* trend^b^						0.001
Occupational activity						
Sedentary	1,288	886	1	1	1	1
Standing	561	433	0.89 (0.77–1.04)	0.91 (0.78–1.06)	1.00 (0.84–1.17)	0.99 (0.83–1.17)
Manual	739	516	0.99 (0.86–1.13)	0.97 (0.84–1.12)	1.14 (0.98–1.34)	1.08 (0.92–1.27)
Heavy manual	64	55	0.80 (0.55–1.16)	0.76 (0.52–1.11)	0.91 (0.61–1.35)	0.90 (0.60–1.37)
Unemployment	557	598	0.64 (0.55–0.74)	0.85 (0.73–0.99)	0.98 (0.83–1.15)	1.05 (0.89–1.26)
Total activity (MET-h/day)						
< 40.0	714	595	1	1	1	1
40.0–44.9	168	129	1.09 (0.84–1.40)	1.07 (0.82–1.38)	1.10 (0.83–1.44)	1.08 (0.81–1.45)
45.0–49.9	171	138	1.03 (0.80–1.32)	0.97 (0.75–1.25)	0.98 (0.75–1.29)	0.90 (0.68–1.19)
≥ 50.0	2,159	1,629	1.10 (0.97–1.25)	1.07 (0.94–1.22)	1.07 (0.92–1.25)	1.02 (0.87–1.20)
*p* trend^b^						0.438

*OR* odds ratio, *CI* 95% confidence interval

^a^Adjusted for alcohol intake (g/day), menopause (yes/no), hormone therapy (HT) use (yes/no), HT duration, number of children, previous benign tumor, age at first birth, smoking (never, previous, current), and education (short education (≤ 7 years), medium education (8–10 years), long education (> 10 years), and mutual adjustment for other physical activities and occupational physical activity (e.g., to investigate the association between sport and MD, we adjusted for cycling, walking, gardening, housework, and do-it-yourself work)

^b^Trend test (Cochran–Armitage) between the levels of physical activities (fully adjusted model^a^)

**Table 3:** Association of physical activity with MD stratified by BMI < 30 (kg/m^2^) and BMI ≥ 30 (kg/m^2^), in Diet, Cancer and Health cohort

	BMI < 30 (kg/m^2^)	BMI ≥ 30 (kg/m^2^)
	OR^a^ (95% CI)	OR^a^ (95% CI)
Physical activity		
No participation in sport	1	1
Participation in sport	1.01 (0.89–1.15)	1.25 (0.92–1.69)
No walking	1	1
Walking	0.90 (0.69–1.16)	0.97 (0.59–1.60)
No cycling	1	1
Cycling	1.03 (0.89–1.19)	0.89 (0.65–1.22)
No gardening	1	1
Gardening	1.02 (0.90–1.17)	1.19 (0.88–1.60)
No do-it-yourself work	1	1
Do-it-yourself work	1.11 (0.97–1.27)	0.94 (0.68–1.28)
No housework	1	1
Housework	0.99 (0.55–1.76)	0.77 (0.22–2.69)

*OR* odds ratio, *CI* 95% confidence interval

^a^Adjusted for alcohol intake (g/day), menopause (yes/no), hormone therapy (HT) use (yes/no), HT duration, number of children, previous benign tumors, age at first birth, smoking (never, previous, current), and education (short education (≤ 7 years), medium education (8–10 years), long education (> 10 years), and mutual adjustment for other physical activities and occupational physical activity (e.g., to investigate the association between sport and MD, we adjusted for cycling, walking, gardening, housework, and do-it-yourself work)

**Table 4:** Association of physical activity with MD stratified by menopausal status in Diet, Cancer and Health cohort

	Premenopausal	Postmenopausal
	OR^a^ (95% CI)	OR^a^ (95% CI)
Physical activity		
No participation in sport	1	1
Participation in sport	1.01 (0.74–1.37)	1.25 (0.92–1.69)
No walking	1	1
Walking	0.98 (0.54–1.77)	0.97 (0.59–1.60)
No cycling	1	1
Cycling	0.90 (0.64–1.27)	0.98 (0.84–1.13)
No gardening	1	1
Gardening	1.25 (0.91–1.70)	1.00 (0.87–1.14)
No do-it-yourself work	1	1
Do-it-yourself work	1.15 (0.84–1.58)	1.04 (0.91–1.20)
No housework	1	1
Housework	0.94 (0.34–2.60)	0.77 (0.22–2.69)

*OR* odds ratio, *CI* 95% confidence interval

^a^Adjusted for alcohol intake (g/day), BMI, waist circumference hormone therapy (HT) use (yes/no), HT duration, number of children, previous benign tumor, age at first birth, smoking (never, previous, current), and education (short education (≤ 7 years), medium education (8–10 years), long education (> 10 years), and mutual adjustment for other physical activities and occupational physical activity (e.g., to investigate the association between sport and MD, we adjusted for cycling, walking, gardening, housework, and do-it-yourself work)

**Table 5:** Association of physical activity with MD stratified by menopausal status and BMI in Diet, Cancer and Health cohort

	Premenopausal		Postmenopausal	
	BMI < 30 (kg/m^2^)	BMI ≥ 30 (kg/m^2^)	BMI < 30 (kg/m^2^)	BMI ≥ 30 (kg/m^2^)
	OR^a^ (95% CI)	OR^a^ (95% CI)	OR^a^ (95% CI)	OR^a^ (95% CI)
Physical activity				
No participation in sport	1.00	1.00	1.00	1.00
Participation in sport	0.89 (0.63–1.26)	1.15 (0.52–2.55)	1.00 (0.86–1.15)	1.07 (0.76–1.53)
No walking	1.00	1.00	1.00	1.00
Walking	1.04 (0.53–2.04)	0.84 (0.19–3.66)	0.85 (0.63–1.14)	1.13 (0.62–2.07)
No cycling	1.00	1.00	1.00	1.00
Cycling	0.90 (0.60–1.35)	1.04 (0.48–2.25)	1.03 (0.88–1.21)	0.77 (0.54–1.11)
No gardening	1.00	1.00	1.00	1.00
Gardening	1.29 (0.91–1.84)	1.44 (0.67–3.11)	0.99 (0.86–1.15)	1.03 (0.73–1.46)
No do-it-yourself work	1.00	1.00	1.00	1.00
Do-it-yourself work	1.13 (0.80–1.60)	1.26 (0.57–2.79)	1.07 (0.92–1.24)	0.92 (0.64–1.32)
No housework	1.00	1.00	1.00	1.00
Housework	0.60 (0.19–1.96)	1.00 (1.00–1.00)	0.82 (0.40–1.68)	0.34 (0.05–2.08)

*OR* odds ratio, *CI* 95% confidence interval

^a^Adjusted for alcohol intake (g/day), BMI, waist circumference hormone therapy (HT) use (yes/no), HT duration, number of children, previous benign tumor, age at first birth, smoking (never, previous, current), and education (short education (≤ 7 years), medium education (8–10 years), long education (> 10 years), and mutual adjustment for other physical activities and occupational physical activity (e.g., to investigate the association between sport and MD we adjusted for cycling, walking, gardening, housework, and do-it-yourself work)

## Discussion

From this large prospective cohort study, we did not detect association between leisure time, transport-related, or occupational physical activity and MD. A positive association between certain leisure time physical activities and MD detected in crude models seemed explained by mainly by BMI and waist circumference. In agreement with our findings, three studies suggested weakly positive association between physical activity and MD in unadjusted analysis, which the association attenuated after adjustment for BMI, reproductive factors, and lifestyle variables [16, 18, 19].

Our finding of no association between physical activity and MD corroborates with the majority of studies on the topic, although the direct comparisons between studies are challenging due to differences in methods for measuring mammographic density and the types of physical activity that were assessed [20, 21, 23–25]. A systematic review of 20 published studies, Yaghjyan et al. found no evidence of association between physical activity and MD when assessing the timing of physical activity (during adolescence, current/recent, past, and lifetime) and women’s menopausal status [25]. In addition, a large study of 1900 women aged 40–93 years did not show any association between physical activity and percent MD in neither pre- or postmenopausal women [23]. It is important to mention that women included in this study had breast cancer; therefore, the result may not be generalizable to all women.

Although majority of studies found no association between physical activity and MD [20, 21, 23–25], few suggested that physical activity may be inversely associated with MD [12, 14, 22]. Gram et al. in 2720 Norwegian women aged 40–56 years found a borderline significant reduction of breast density with high-risk MD pattern assessed by Tabar classification who were highly active as compared with inactive women [12]. In this study, non-significant inverse associations were detected for the total population of 2720 women and in premenopausal women for vigorous physical activity, occupational physical activity, and leisure time physical activity. Lopez et al. investigated the effect of physical in activity and percent MD among 294 Hispanic women older than 40 years, and found association between physical inactivity per day and higher percent MD [14]. Thrih et al. in a Swedish cross-sectional study of 38,913 women aged 40–74 years, found that women with high physical activity had lower breast absolute dense volume compared to women with the lowest physical activity level [22].

In contrast to our results on no association between physical activity and MD in obese women (BMI ≥ 30 kg/m^2^), three previous studies reported a statistically significant inverse association between physical activity and mammographic density in women with high BMI [13, 15, 17]. Qureshi et al. in Norwegian cohort of 2218 postmenopausal women aged 50–69 years found evidence of an inverse association between mammographic percent density and physical activity in overweight women [17]. In a longitudinal study of 2000 Mediterranean women, Masala et al. found that there was an inverse association between leisure time physical activity and MD among postmenopausal overweight/obese women [15]. A cross-sectional study of 474 US women who reported their physical activity a year before their diagnosis with breast cancer, found no association between physical activity and neither mammographic percent density nor dense area. However, a statistical significant reduction of mammographic percent density and dense area was observed among obese postmenopausal women with higher levels of sport/recreational physical activity [13]; however, their results may not be generalizable to cancer-free women.

Physical activity has been proposed to influence breast cancer risk through several mechanisms, including weight loss, obesity prevention, reduce sex hormones, insulin, and insulin-like growth factor levels, and induce immune system function and mechanism of DNA repair [8]. However, the biological mechanism by which physical activity may affect MD remains unsolved. Previous findings have shown that physical activity could reduce circulating levels of and cumulative exposure to sex steroid hormones during the premenopausal period [41]. In addition, physical activity has been shown to decrease estrogen levels among postmenopausal women, in part by affecting adipose tissue reduction [42, 43]. During the postmenopausal period, adipose tissue is the main source of estrogen biosynthesis.

Previous studies suggested that physical activity may indirectly affect MD by decreasing the estrogen levels production in peripheral tissue among postmenopausal women [42, 43].

During the postmenopausal when the ovaries do not produce estrogen, estrogens are primarily produced from the conversion of androgens by aromatase enzyme in adipose tissue [44]. Higher levels of estrogen have been linked to high MD during pre- and postmenopausal women [45, 46]. Furthermore, a study of postmenopausal women detected significant inverse association between increasing activity level and circulating testosterone and estradiol and significant positive association with sex hormone-binding globulin [47]. The lack of an overall association between physical activity and MD in the current and previous studies [20, 21, 23–25] implies that, physical activity influences breast cancer risk through other mechanisms than breast density.

Major strength of this study was the large, prospective cohort of Danish women with well-defined information on physical activity, determinants of MD, breast cancer risk factors, and possibility of linkage to the Mammography Registry with validated data on MD. This is one of the largest studies to date examining the association between physical activity and MD. We furthermore benefited from detailed data on different types of physical activity including leisure time, transport-related (cycling), and occupational physical activity, while the majority of other studies assessed non-occupational leisure time physical activity only. We also had high proportion of physical active women in the cohort (47.6%), allowing for statistical power to examine associations within various subgroups of physical activity.

There are some limitations in this study that should be considered. We utilized a Danish-dichotomized MD score as mixed/dense or fatty breasts which can measure only large effects on MD, as no other measure of MD was available. However, this dichotomous outcome has been utilized in earlier studies of MD and breast cancer mortality [34], early childhood BMI and MD [35], active tobacco smoking and MD [36], alcohol use and MD [38], air pollution and MD [37], and HRT use and MD and breast cancer risk [39]. Furthermore, we have successfully validated dichotomous MD measure and found good agreement with BI-RADS [33]. In addition, information on physical activity was not updated after baseline, which might induce some misclassification, but since the average time between cohort baseline and mammographic screening used for MD was 1 year it should not distort the results. Another weakness in this study is the lack of longitudinal data on both physical activity and MD measurements which would facilitate the optimal design to study whether change in physical activity is associated with MD change. Finally, in this study, we included women at age of 50 years, at which most women in this age experienced decline in MD; however, in the analyses we accounted for menopausal status and we included both pre- and postmenopausal women. Considering the lack of data on MD change and utilization of crude measure of MD, both of which would likely bias our results toward to null finding. However, since our findings corroborate with majority of studies on this topic, this confirms that, despite limitations in our design, we contribute with novel evidence that physical activity is not a determinant of MD.

In conclusion, in this cohort of women aged 50 years and above, we found no evidence of association between physical activity and MD. Future studies with detailed information on physical activity over a longer period are needed to confirm our finding that physical activity influences breast cancer risk independently from MD.

## Electronic supplementary material

Below is the link to the electronic supplementary material.

Supplementary material 1 (DOCX 31 KB)

Supplementary material 2 (DOCX 14 KB)

## Abbreviations

MD: Mammographic density
DCH: Danish Diet, Cancer and Health cohort
OR: Odds ratio
CI: Confidence intervals
BMI: Body mass index
BI-RADS: Breast Imaging Reporting and Data System
