# Supplementary material for: Regular physical activity and mammographic density: a cohort study
Source: Cancer Causes Control. 2018 Sep 7;29(11):1015–25. doi: 10.1007/s10552-018-1075-3 (PMC6245045; doi:10.1007/s10552-018-1075-3)
Supplement: Supplementary file 1 — Supplementary material 1 (DOCX 31 KB) [file 10552_2018_1075_MOESM1_ESM.docx]

**Regular physical activity and mammographic density: a cohort study**

Cancer Cause and Control

Shadi Azam^1^*, Katja Kemp Jacobsen^2^, Arja R.Aro^1^, My von Euler-Chelpin^3^, Anne Tjønneland^4^, Ilse Vejborg^5^, Elsebeth Lynge^3^, Zorana J. Andersen^3^

^1^Unit for Health Promotion, Department of Public Health, University of Southern Denmark, Niels Bohrs Vej 9, 6700 Esbjerg, Denmark;

^2^Department of Technology, Faculty of Health and Technology, University College Copenhagen, Denmark;

^3^Section of Environmental Health, Department of Public Health, University of Copenhagen, Denmark;

^4^ Danish Cancer Society Research Center, Danish Cancer Society, Denmark;

^5^Department of Radiology and Diagnostic Imaging Centre, Copenhagen University Hospital, Rigshospitalet, Denmark.

Shadi Azam ([shadi.azam@ki.se](mailto:shadi.azam@ki.se))

**Online source 2**. Association of physical activity with MD among 5,703 women in Diet, Cancer and Health cohort

|  | Mixed/dense  MD | Fatty  MD | Crude | Age Adjusted | Fully Adjusted^a^ | Fully adjusted^a^ (+BMI and waist circumference) |
| --- | --- | --- | --- | --- | --- | --- |
|  |  |  | RR (95% CI) | RR (95% CI) |  |  |
| Physical activity |  |  |  |  |  |  |
| No participation in sport  Participation in sport | 1,627  1,582 | 1,360  1,128 | 1.00  1.07 (1.02-1.12) | 1.00  1.07 (1.02-1.12) | NA | NA |
| No walking  Walking | 225  2,984 | 189  2,299 | 1.00  1.04 (0.95-1.14) | 1.00  1.02 (0.93-1.11) |  |  |
| No cycling  Cycling | 906  2,306 | 794  1,697 | 1.00  1.08 (1.03-1.14) | 1.00  1.04 (0.99-1.09) |  |  |
| No gardening  Gardening | 1,512  1,700 | 1,212  1,279 | 1.00  1.03 (0.98-1.08) | 1.00  1.03 (0.98-1.07) |  |  |
| No do-it-yourself work  Do-it-yourself work | 1,876  1,333 | 1,596  892 | 1.00  1.11 (1.06-1.16) | 1.00  1.07 (1.02-1.11) |  |  |
| No housework  Housework | 43  3,166 | 39  2,449 | 1.00  1.08 (0.87-1.32) | 1.00  0.99 (0.82-1.21) |  |  |
| Physical activity h/week  Participation in sport  No activity  0.5-2.0  2.0-4.0  ≥ 4  p-trend^b^ | 1,627  800  546  236 | 1,360  572  379  177 | 1.00  1.07 (1.01-1.13)  1.08 (1.01-1.15)  1.11 (0.90-1.37) | 1.00  1.06 (1.01-1.12)  1.08 (1.01-1.14)  1.05 (0.97-1.15) |  |  |
| Walking  No activity  0.5-2.0  2.0-4.0  ≥ 4  p-trend^b^ | 225  522  897  1565 | 189  374  669  1256 | 1.00  1.07 (0.97-1.19)  1.05 (0.96-1.16)  1.02 (0.93-1.12) | 1.00  1.03 (0.93-1.14)  1.02 (0.93-1.12)  1.01 (0.92-1.10) |  |  |
| Cycling  No activity  0.5-2.0  2.0-4.0  ≥ 4  p-trend^b^ | 906  634  673  999 | 794  433  494  770 | 1.00  1.11 (1.04-1.19)  1.08 (1.01-1.16)  1.06 (0.99-1.12) | 1.00  1.07 (1.01-1.15)  1.05 (1.01-1.15)  1.00 (0.95-1.06) |  |  |
| Gardening  No activity  0.5-2.0  2.0-4.0  ≥ 4  p-trend^b^ | 1,512  724  481  495 | 1,212  504  372  403 | 1.00  1.06 (1.00-1.13)  1.02 (0.95-1.09)  0.99 (0.93-1.06) | 1.00  1.04 (0.98-1.09)  1.02 (0.96-1.09)  1.01 (0.94-1.08) |  |  |
| Do-it yourself work  No activity  0.5-2.0  2.0-4.0  ≥ 4  p-trend^b^ | 1,876  789  325  219 | 1,596  513  225  154 | 1.00  1.12 (1.06-1.18)  1.09 (1.01-1.18)  1.09 (0.99-1.19) | 1.00  1.07 (1.02-1.13)  1.05 (0.98-1.13)  1063 (0.97-1.15) |  |  |
| Housework  No activity  0.5-2.0  2.0-4.0  ≥ 4  p-trend^b^ | 43  141  814  2,211 | 39  96  582  1,771 | 1.00  1.13 (0.90-1.43)  1.11 (0.90-1.37)  1.06 (0.86-1.30) | 1.00  1.05 (0.84-1.30)  1.01 (0.83-1.24)  0.98 (0.81-1.19) |  |  |
| Occupational activity  Sedentary  Standing  Manual  Heavy manual  Unemployment | 1,288  561  739  64  557 | 886  433  516  55  598 | 1.00  0.95 (0.89-1.01)  0.99 (0.94-1.05)  0.91 (0.77-1.07)  0.81 (0.76-0.87) | 1.00  0.96 (0.90-1.02)  0.98 (0.92-1.04)  0.87 (0.74-1.03)  0.93 (0.87-0.99) |  |  |
| Total activity (MET-hours/day)  <40.0  40.0-44.9  45.0-49.9  ≥50.0  p-trend^b^ | 714  168  171  2,159 | 595  129  138  1,629 | 1.00  1.04 (0.93-1.16)  1.01 (0.91-1.13)  1.04 (0.99-1.11) | 1.00  1.03 (0.93-1.15)  0.99 (0.89-1.10)  1.03 (0.98-1.09) |  |  |

RR; Relative Risk, CI : 95% confidence interval; a: adjusted for alcohol intake (g/day), menopause (yes/no), hormone therapy (HT) use (yes/no), HT duration, number of children, previous benign tumor, age at first birth, smoking (never, previous, current), and education (short education (≤ 7 years), medium education (8-10 years), long education (> 10 years), and mutual adjustment for other physical activities and occupational physical activity (e.g., to investigate the association between sport and MD we adjusted for cycling, walking, gardening, housework and do-it-your-self work),
