# Supplementary material for: Regular physical activity and mammographic density: a cohort study
Source: Cancer Causes Control. 2018 Sep 7;29(11):1015–25. doi: 10.1007/s10552-018-1075-3 (PMC6245045; doi:10.1007/s10552-018-1075-3)
Supplement: Supplementary file 2 — Supplementary material 2 (DOCX 14 KB) [file 10552_2018_1075_MOESM2_ESM.docx]

**Regular physical activity and mammographic density: a cohort study**

Cancer Cause and Control

Shadi Azam^1^*, Katja Kemp Jacobsen^2^, Arja R.Aro^1^, My von Euler-Chelpin^3^, Anne Tjønneland^4^, Ilse Vejborg^5^, Elsebeth Lynge^3^, Zorana J. Andersen^3^

^1^Unit for Health Promotion, Department of Public Health, University of Southern Denmark, Niels Bohrs Vej 9, 6700 Esbjerg, Denmark;

^2^Department of Technology, Faculty of Health and Technology, University College Copenhagen, Denmark;

^3^Section of Environmental Health, Department of Public Health, University of Copenhagen, Denmark;

^4^ Danish Cancer Society Research Center, Danish Cancer Society, Denmark;

^5^Department of Radiology and Diagnostic Imaging Centre, Copenhagen University Hospital, Rigshospitalet, Denmark.

Shadi Azam ([shadi.azam@ki.se](mailto:shadi.azam@ki.se))

Online source 1. Characteristics of the 5,356 study participants compared to 24,519 non-participants (The remaining participants in the Danish Diet, Cancer and Health cohort)

|  | Included in this study | Not included |
| --- | --- | --- |
| DCH participants | 5,356 | 24,519 |
| Age (years), mean (SD) | 56.2 (4.5) | 53.3 (4.4) |
| Parous (%) | 4,544 (84.8) | 22,416 (92.0) |
| HT use (ever), n (%) | 2,518 (47.0) | 10,637 (43.5) |
| BMI (kg/m2), mean (SD)  BMI <25 (kg/m2), n (%)  BMI ≥25 (kg/m2), n (%) | 25.9 (4.7)  2,624 (49.0)  2,732 (51.0) | 25.5 (4.3)  12,850 (52.4)  11,669 (47.5) |
| Physically active | 2,572 (48.0) | 14,875 (60.7) |
| Short education (≤ 7 years), n (%)  Medium education (8-10 years), n (%)  Long education (> 10 years), n (%) | 1,906 (35.6)  2,624 (49.0)  826 (15.4) | 7,443 (30.7)  12,193 (50.3)  4,606 (19.0) |
